# Hypertension outcomes of adrenalectomy in patients with primary aldosteronism: a systematic review and meta-analysis

**DOI:** 10.1186/s12902-017-0209-z

**Published:** 2017-10-03

**Authors:** Yu Zhou, Meilian Zhang, Sujie Ke, Libin Liu

**Affiliations:** 1 Department of Endocrinology, Fujian Medical University Union Hospital, Fuzhou, Fujian 350001 China; 2 Department of Ultrasonography, Fujian Medical University Union Hospital, Fuzhou, Fujian 350001 China

**Keywords:** Adrenalectomy, Hypertension, Primary aldosteronism, Meta-analysis, Review

## Abstract

**Background:**

The hypertension cure rate of unilateral adrenalectomy in primary aldosteronism (PA) patients varies widely in existing studies.

**Methods:**

We conducted an observational meta-analysis to summarize the pooled hypertension cure rate of unilateral adrenalectomy in PA patients. Comprehensive electronic searches of PubMed, Embase, Cochrane, China National Knowledge Internet (CNKI), WanFang, SinoMed and Chongqing VIP databases were performed from initial state to May 20, 2016. We manually selected eligible studies from references in accordance with the inclusion criteria. The pooled hypertension cure rate of unilateral adrenalectomy in PA patients was calculated using the DerSimonian–Laird method to produce a random-effects model.

**Results:**

Forty-three studies comprising approximately 4000 PA patients were included. The pooled hypertension cure rate was 50.6% (95% CI: 42.9–58.2%) for unilateral adrenalectomy in PA. Subgroup analyses showed that the hypertension cure rate was 61.3% (95% CI: 49.4–73.3%) in Chinese studies and 43.7% (95% CI: 38.0–49.4%) for other countries. Furthermore, the hypertension cure rate at 6-month follow-up was 53.3% (95% CI: 36.0–70.5%) and 49.6% (95% CI: 40.9–58.3%) for follow-up exceeding 6 months. The pooled hypertension cure rate was 50.9% (95% CI: 40.5–61.3%) from 2001 to 2010 and 50.2% (95% CI: 39.0–61.5%) from 2011 to 2016.

**Conclusions:**

The hypertension cure rate for unilateral adrenalectomy in PA is not optimal. Large clinical trials are required to verify the utility of potential preoperative predictors in developing a novel and effective prediction model.

## Background

Primary aldosteronism (PA) is a group of disorders in which aldosterone production is inappropriately high for sodium status, relatively independent of the major regulators of secretion (e.g., angiotensin II, plasma potassium concentration), and is not suppressed by sodium loading [1]. Some studies have reported that PA is present in >5% of hypertensive patients and may exceed 10% [2–4]. PA is typically caused by an adrenal adenoma and unilateral or bilateral adrenal hyperplasia. However, in a small proportion of cases, adrenal carcinoma and familial hyperaldosteronism can also precipitate PA.

Idiopathic hyperaldosteronism (IHA) should be medically treated with a mineralocorticoid receptor antagonist, whereas unilateral primary aldosteronism, which includes aldosterone-producing adenoma (APA) and unilateral adrenal hyperplasia (UAH), is curable by surgical intervention [5]. However, after unilateral adrenalectomy, not all PA patients are completely cured of their hypertension, despite normalization of the biochemical marker abnormalities. After adrenalectomy for unilateral primary aldosteronism, complete hypertension cure rate and the factors associated with complete hypertension cure are variable. Previous studies have reported hypertension cure rates ranging from 35% to 63.8%, with intrinsic risk factors being identified. These include age, gender, duration of hypertension, body mass index (BMI), and preoperative prescribing of antihypertensive agents [6–9]. Furthermore, a meta-analysis of the 25 studies with 1685 patients showed that the pooled proportion of normotension following adrenalectomy is 52% [10]. However, the study selects 25 articles which 4 Chinese studies only. A multicenter epidemiologic study in 11 provinces of China between January 2010 and October 2011 reveals that PA prevalence is 7.1% among 1656 patients with resistant hypertension [11]. Given the high prevalence, PA patients in China are more and more interested in solving the problem of hypertension by adrenalectomy. Consequently, in the present study, we added the Chinese-language publication data and established more strict inclusion criteria to more comprehensively estimate the complete hypertension cure rate of unilateral adrenalectomy in PA patients.

## Methods

### Literature search strategy

Online retrieval of the following databases was conducted: PubMed, Embase, Cochrane, China National Knowledge Internet (CNKI), WanFang, SinoMed and Chongqing VIP. Eligible studies were manually selected from references according to the inclusion criteria. The search date was from initial state to May 20, 2016. The following keywords were used to identify potentially relevant studies from all databases: ‘hyperaldosteronism’ and ‘adrenalectomy’. We also retrieved the references from all relevant publications to obtain further salient studies. Two independent reviewers (Yu Zhou and Mei-Lian Zhang) conducted the literature screen of relevant titles and abstracts according to inclusion and excluding criteria. Su-jie Ke was the adjudicator for any articles that were disputed.

### Inclusion and exclusion criteria

The following criteria were used for screening of the literature: (1) all PA patients to undergo unilateral adrenalectomy and sample size should be ≥20; (2) study design comprised case-control and cohort studies; (3) the study provided the complete hypertension cure rate of unilateral adrenalectomy, or sufficient data for derivation of the pooled hypertension cure rate; (4) hypertension cure criteria was defined as normal blood pressure (systolic blood pressure (SBP) <140 mmHg and diastolic blood pressure (DBP) <90 mmHg) without requirement for antihypertensive medications; (5) each study should provide a clear follow-up period; (6) the study was written in English and Chinese. Those studies which were not eligible for inclusion were excluded. If multiple published reports were from the same study cohort, we only included the study with the most detailed information and sample size.

### Extraction of data

All data were extracted separately by the two aforementioned investigators. In the event of a dispute between the investigators, a discussion was conducted in order to arrive at consensus. Information obtained from each study included: author’s first name, year of publication, country, years of inclusion, follow-up time, patients cured, patients followed up, cure rate (%), pathological results, and study type.

### Statistical analysis

This study was conducted and reported according to the recommendation of the Meta-analysis Of Observational Studies in Epidemiology (MOOSE) group [12]. Heterogeneity between the studies was estimated by calculating the *I* ^2^ statistic, which shows the percentage of variation between studies due to heterogeneity rather than by chance. *I* ^2^ < 25% is considered low, 25–50% considered moderate, and >50% is regarded as high-level heterogeneity [13, 14]. Given the high heterogeneity between studies, we used the DerSimonian and Laird method in generating the random effects models for the pooled estimation of prevalence. We conducted an analysis of subgroups to explore the potential sources of heterogeneity, including country (China and Other), follow-up time (≥6 months and <6 months), number of patients followed up (≥50 and <50), Publication Year (2001–2010 and 2011–2016), and pathology (APA Only and Other). Sensitivity analyses were conducted by excluding prospective studies. Funnel plots and the Egger test were used to test for publication bias. A *P* value of less than 0.05 was considered statistically significant. All statistical calculations were performed using STATA, version 12 (STATA, College Station, TX). Ethics approval was not required as this was a secondary, literature-based study.

## Results

### Literature search

**Fig. 1:**
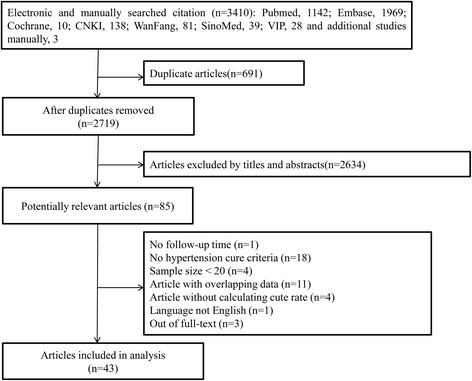
Flow diagram of included/excluded studies

The literature search process is depicted in Fig. 1. The literature search initially yielded a total of 3410 articles, of which 691 duplicates were excluded and 2634 studies were removed after reviewing titles and abstracts. Thus, 85 studies were chosen for full-text assessment and critical appraisal. We excluded the cited articles for the following reasons: no follow-up period (*n* = 1); no hypertension cure criteria (*n* = 18); sample size <20 (*n* = 4); article with overlapping data (*n* = 11); article with no calculation of cure rate (n = 4); non-English language (n = 1); Out of full-text (*n* = 3). Finally, there were 43 studies included in our meta-analyses [6–9, 15–53].

### Study characteristics

The main characteristics from the included studies are shown in Table 1. The relevant number of patients followed up ranged from 20 to 376, with a total sample size of 3776. All studies were in Chinese and English. Studies were carried out in different countries, including the USA, China, Japan, Italy, Sweden, Germany, Australia, Czech Republic, Singapore and France. Forty of the included studies were methodologically retrospective. In contrast, only three studies were prospective cohort studies. All the cases were pathologically confirmed postoperatively.

**Table 1:** Characteristics of the Included Studies

Study,Year	Country	Years of Inclusion	Follow-up Time	Patients Cured	Patients Followed	Cure Rate(%)	Pathology	Study Type
Horita et al. 2001 [15]	Japan	1977–1999	>6 months	13	26	50.0	APA	retrospective
Sawka et al. 2001 [16]	America	1993–1999	0.1–77.9 months	31	93	33.3	APA + Other	retrospective
Fukudome et al. 2002 [17]	Japan	1976–1998	1.2–23.1 years	34	46	73.9	APA	retrospective
Rossi et al. 2002 [18]	America	1994–2000	1-63 months	20	30	66.7	APA + Other	retrospective
Tan et al. 2002 [19]	China	1984–2001	>3 months	44	46	95.7	APA + Other	retrospective
Goh et al. 2004 [20]	Singapore	1996–2002	1-60 months	23	46	50.0	APA + Other	retrospective
Meyer et al. 2005 [21]	Germany	1988–2001	38-134 months	8	24	33.3	APA + Other	retrospective
Omura et al.2006 [6]	Japan	1995–2005	1 year	44	69	63.8	APA	retrospective
Han et al.2006 [22]	China	2003–2005	3-37 months	35	47	74.5	APA + Other	retrospective
Pang et al.2007 [23]	Australia	1995–2005	>1 month	18	53	34.0	APA + Other	prospective
Ziaja et al.2007 [24]	Poland	1995–2005	1-10 years	10	31	32.3	APA + Other	retrospective
Walz et al.2008 [25]	Germany	1994–2007	>1 years	48	160	30.0	APA + Other	prospective
Zarnegar et al.2008 [7]	America	1994–2005	>6 months	35	100	35.0	APA + Other	retrospective
White et al.2008 [26]	America	1996–2007	0.1–96.7 months	38	54	70.4	APA + Other	retrospective
Mourad et al.2008 [27]	France	1997–1999	30-56 months	23	58	39.7	APA	retrospective
Wu et al.2009 [8]	China	1999–2007	>1 year	95	150	63.3	APA	retrospective
Chiou et al.2009 [28]	China	1987–2006	>6 months	18	51	35.3	APA	retrospective
Campagnacci et al.2009 [29]	Italy	1994–2006	20-128 months	21	50	42.0	APA	retrospective
Wang et al.2010 [30]	China	2002–2007	1.2–5.3 years	54	93	58.1	APA	retrospective
Tresallet et al.2010 [31]	France	1997–2008	>6 months	33	57	57.9	APA + Other	retrospective
Mathur et al.2010 [32]	America	NA	1 month-9 year	17	85	20.0	APA + Other	retrospective
Kim et al.2010 [33]	Korea	1995–2008	6-159 months	16	27	59.3	APA	retrospective
Fu et al.2011 [34]	China	2000–2004	>1 years	61	212	28.8	APA	prospective
Waldmann et al.2011 [35]	Germany	1993–2009	>1 years	17	30	56.7	APA + Other	retrospective
Tang et al.2011 [36]	China	1999–2009	6 months-2 years	180	227	79.2	APA + Other	retrospective
Wang et al.2011 [37]	China	2008–2010	1 week-1 year	10	25	40.0	APA + Other	retrospective
Linden et al.2012 [38]	France	2001–2009	6-12 months	68	156	43.5	APA + Other	retrospective
Wang et al.2012 [39]	China	2008–2010	>6 months	43	83	51.8	APA + Other	retrospective
Wang et al.2012 [40]	China	2009–2011	6-18 months	17	20	85.0	APA + Other	retrospective
Wang et al.2012 [41]	China	2002–2009	>6 months	44	82	53.7	APA	retrospective
Zhang et al.2013 [42]	China	2005–2011	>6 months	207	376	55.1	APA	retrospective
Aronova et al.2014 [43]	America	2004–2013	>1 years	21	47	44.7	APA	retrospective
Jiang et al.2014 [44]	China	2004–2011	15-110 months	88	164	53.7	UAH	retrospective
Lim et al.2014 [45]	China	1993–2011	1.9–11.7 years	127	133	95.4	APA + Other	retrospective
Wachtel et al.2014 [46]	America	1997–2013	>1 years	13	85	15.3	APA + Other	retrospective
Hartmann et al.2014 [47]	Czech Republic	2001–2011	1 year	17	51	33.3	APA + Other	retrospective
Utsumi et al.2014 [48]	Japan	1995–2012	>6 months	56	132	42.4	APA + Other	retrospective
Wolley et al.2015 [49]	Australia	2000–2014	6-24 months	29	80	36.3	APA + Other	retrospective
Xie et al.2015 [9]	China	2009–2014	0.17-5 years	43	94	45.7	APA	retrospective
Hu et al.2015 [50]	China	2009–2014	>1 years	29	46	63.0	APA + Other	retrospective
Citton et al.2015 [51]	Italy	1990–2013	1 month	67	122	54.9	APA + Other	retrospective
Volpe et al.2015 [52]	Sweden	1985–2010	0.5-26 years	46	120	38.3	APA + Other	retrospective
Fujita et al.2016 [53]	Japan	2000–2015	>1 years	36	95	37.9	APA + Other	retrospective

### Hypertension cure rate of unilateral adrenalectomy in PA

**Fig. 2:**
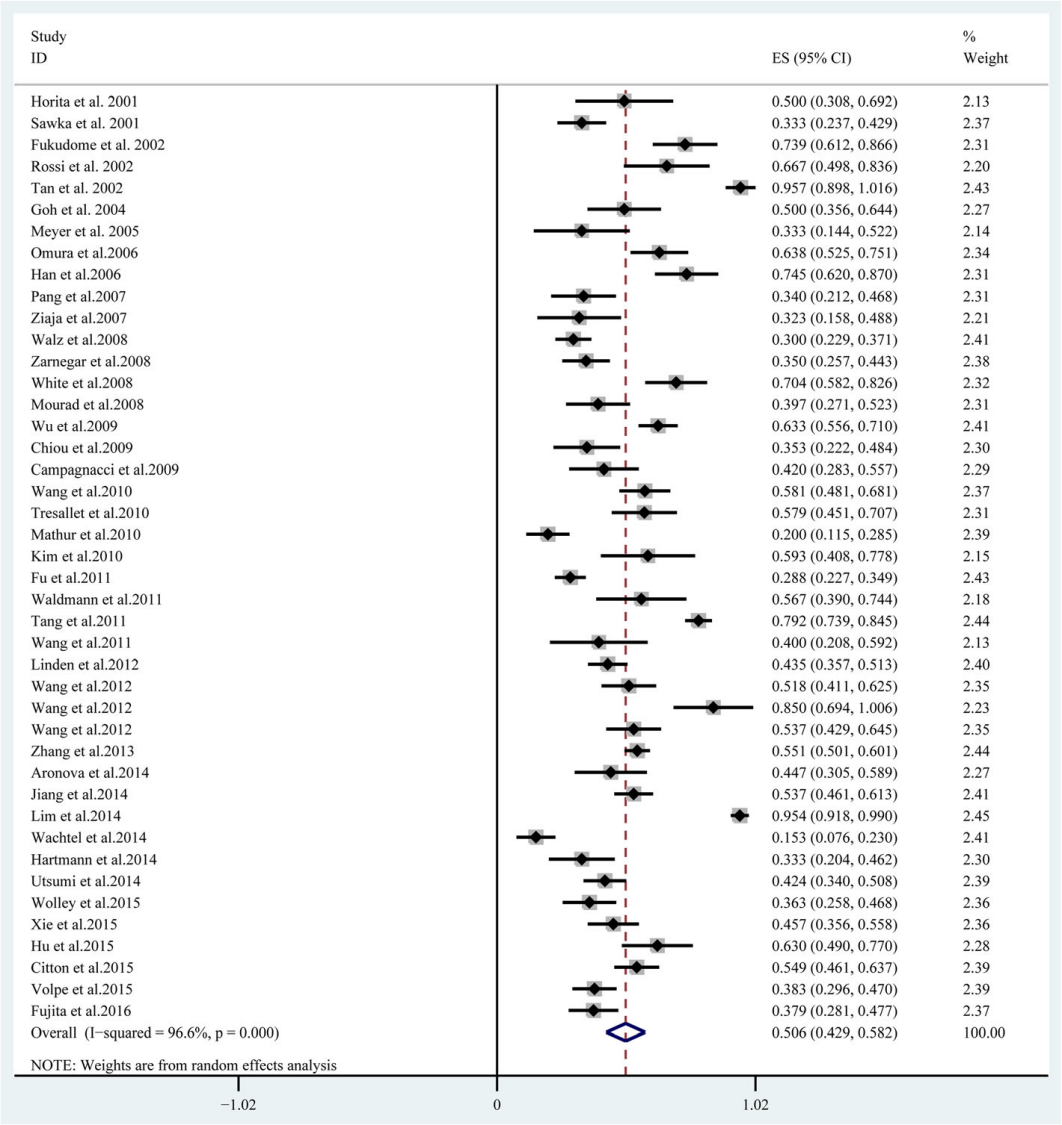
Forest plot of hypertension cure rate of unilateral adrenalectomy for all PA patients

Unilateral adrenalectomy in PA resulted in an overall hypertension cure rate that ranged between 15.0% and 96.0%. The overall meta-analysis cure rate was 50.6% (95% CI: 42.9–58.2%; Fig. 2). The hypertension cure rate of unilateral adrenalectomy in PA also indicated a high level of heterogeneity between the studies (I^2^ = 96.6%, *P* < 0.0001).

The population of PA can be further stratified according to country (China and Other), follow-up time (≥6 months and <6 months), patients followed up (≥50 and <50), Publication Year (2001–2010 and 2011–2016), and pathology (APA Only and Other). The pooled hypertension cure rate of these subgroups is presented in Table 2. In the country setting, the hypertension cure rate of unilateral adrenalectomy in PA in China was 61.3% (95% CI: 49.4–73.3%) from 1849 patients. The pooled cure rate from other countries (43.7%, 95% CI: 38.0–49.4%) was significantly lower than that reported in China (P for subgroup difference < 0.0001). The hypertension cure rate of unilateral adrenalectomy for those followed up over a period of <6 months (53.3%, 95% CI: 36.0–70.5%) was higher than the group followed up over a period of ≥6 months (49.6%, 95% CI: 40.9–58.3%). The cure rate in the number of patients followed up being <50 (59.5%, 95% CI: 46.9–72.1%) was significantly higher than that of the ≥50 group (46.6%, 95% CI: 37.4–55.7%). The pooled hypertension cure rate did not change significantly over time. The pooled cure rate was 50.9% (95% CI: 40.5–61.3%) from 2001 to 2010, which was very similar to the rate of 50.2% (95% CI: 39.0–61.5%) from those studied in 2011 to 2016. In the postoperative pathology setting, there was no significant difference in the APA Only group (50.9%, 95% CI: 43.5–58.2%) and the Other group (50.4%, 95% CI: 39.9–60.8%). The aforementioned subgroups had a high level of heterogeneity.

**Table 2:** Hypertension cure rate of unilateral adrenalectomy by different categories

Category	Subgroup	NO. of Studies	Cute Rate (95% CI) (%)	*N*	I^2^ (%)	*P*
Country	China	16	0.613 (0.494–0.733)	1849	97.5	<0.0001
	Other	27	0.437 (0.380–0.494)	1972	85.9	<0.0001
Follow-up Time	≥6 months	32	0.496 (0.409–0.583)	3081	96.7	<0.0001
	<6 months	11	0.533 (0.360–0.705)	695	96.6	<0.0001
Patients Followed	》50	29	0.466 (0.374–0.557)	3285	97.2	<0.0001
	<50	14	0.595 (0.469–0.721)	491	91.3	<0.0001
Publication Year	2001–2010	22	0.509 (0.405–0.613)	1396	94.9	<0.0001
	2011–2016	21	0.502 (0.390–0.615)	2380	97.5	<0.0001
Pathology	APA Only	14	0.509 (0.435–0.582)	1381	86.3	<0.0001
	Other	29	0.504 (0.399–0.608)	2395	97.4	<0.0001

### Publication bias and sensitivity analysis

**Fig. 3:**
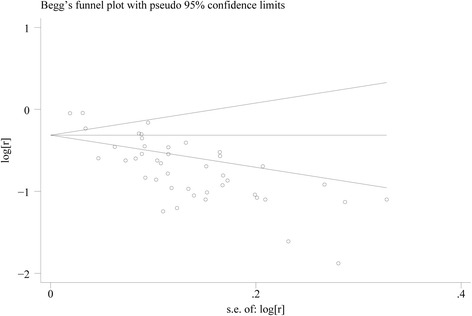
Begg funnel plot for hypertension cure rate of unilateral adrenalectomy

From visual examination of the funnel plots, considerable publication bias was evident (Fig. 3). Probability for the Egger test was less than 0.001. The sensitivity analysis was conducted by excluding the three prospective cohort studies and the results remained the same.

## Discussion

Two reviews [38, 54] showed that the pooled cure rate of complete hypertension after unilateral adrenalectomy was 42% and 41%, respectively. However, these reviews only included partial studies comprising at least 50 consecutive patients, and the potential sources of high-level heterogeneity could not be further explored. In addition, the inclusion of such studies can result in ill-defined postoperative hypertension cure criteria. Thus we performed a systematic evaluation of published studies on the hypertension cure rate of unilateral adrenalectomy in PA patients.

Compared to patients with essential hypertension, those with PA have been shown to have more frequent cardiovascular events, including myocardial infarction, stroke, and atrial fibrillation [55, 56]. This higher incidence may be associated with aldosterone-driven excess left ventricular hypertrophy (LVH), myocardial fibrosis and endothelial dysfunction in PA patients [57–59]. Localization of the source of excessive aldosterone secretion is critical in guiding the management of PA. A biochemical cure with normalization of hypokalemia and aldosterone levels is almost certain following adrenalectomy [7], however, not all patients are completely cured of hypertension after adrenalectomy. This suggests that blood pressure alone is probably not a good outcome of interest in PA studies when viewed in isolation. Complete cure of hypertension as a binary benefit/no benefit variable is an oversimplification. Many patients would perceive achieving control of blood pressure and a large reduction in medications as worthwhile. Additional quality of life benefits have been reported, as have very recent metabolic effects.

In PA patients, surgical treatment is associated with quicker resolution of hypertension, shorter length of hospital stay, and decreased cumulative costs compared with lifelong medical therapy [11]. Most PA patients want a definitive prognosis regarding postoperative probability of hypertension cure when they consider surgical intervention. If such a prognosis could be calculated more precisely, surgeons would be able to explain postoperative hypertension outcome to PA patients with more confidence. In our meta-analysis, a total of 43 studies encompassing approximately 4000 PA patients were included. We found a pooled hypertension cure rate of 50.6% (95% CI: 42.9–58.2%) for unilateral adrenalectomy in PA patients. For different subgroups, the hypertension cure rate of unilateral adrenalectomy in PA was within the range of 43.7% to 61.3%. In the country setting, the pooled hypertension cure rate of unilateral adrenalectomy in PA reported by other countries was lower than that reported in China, most likely caused by the different extent in postoperative follow-up. Wachtel [46] demonstrated that the cure rate at long-term follow-up (≥12 months after surgery) was different from that of short-term follow-up (6–12 months after surgery). This may be associated with a slower return to physiologically normal state. Our study confirmed this: the hypertension cure rate of unilateral adrenalectomy for a follow-up time < 6 months was higher than that for patients followed up over a period of ≥6 months. We also found that sample size affected the hypertension cure rate. The cure rate in the number of patients followed being <50 was higher than that for the number of patients ≥50. The difference between the 2 periods (2001–2011 and 2011–2016) could be explained by an analysis of the type of imaging method used for the diagnostic and tree use of catheterism for adrenal vein samplings (AVS).

Given that the hypertension cure rate of unilateral adrenalectomy was not particularly high, it is necessary to preoperatively forecast hypertension outcome of unilateral adrenalectomy in PA patients. In an effort to more appropriately select candidates for surgery, previous studies have suggested a number of potential preoperative predictors for postoperative hypertension cure in PA patients. These include age, gender, BMI, duration of hypertension, family history of hypertension, preoperative number of antihypertensive agents and others. However, several studies have found no association with hypertension cure for each of the aforementioned predictors. Zarnegar et al. [7]. developed the Aldosteronoma Resolution Score (ARS), which was composed of 4 predictors (number of antihypertensive drugs, BMI, duration of hypertension, and gender), to predict hypertension outcome of adrenalectomy in PA patients. Furthermore, Steichen et al. [54] pointed out that the ARS models were the only prediction model validated to date, and provided only a weak prediction of hypertension cure in individual patients. According to the ARS models, even if none of these features was present in an individual patient, the patient still had a 25% probability of being completely cured by adrenalectomy. In this systematic review, due to the inconsistent data pertaining to risk factors and outcome, we could not obtain a pooled odds ratio through logistic regression analysis in our meta-analysis. Consequently, this hindered us from actually developing a new prediction model of hypertension cure rate of unilateral adrenalectomy. In follow-up studies, large-sample clinical trials are still required to verify these potential preoperative predictors and their utility. In doing so, an effective prediction model of hypertension cure rate of unilateral adrenalectomy will be created.

Although this meta-analysis includes more studies and studies with larger sample sizes than individual studies, there were some limitations of this meta-analysis that might affect the outcome. First, the study was limited to articles that were published in English and Chinese. In addition, the unpublished studies that were not included may have caused a potential bias. It is possible that this could have resulted in the exclusion of several articles pertaining to the hypertension cure rate of unilateral adrenalectomy. Secondly, this research does have publication bias. Thirdly, selection bias of PA patients cannot be fully excluded, resulting from the inclusion of the method of diagnosis of unilateral disease. A part of PA patients don’t be performed adrenalectomy for want of a clear diagnosis. For another, cure of hypertension is likely to depend at least in part on what is left in the remaining adrenal. Thus high-precision imaging and AVS should be used to improve diagnostic accuracy. Fourthly, most original studies do not report post-operative aldo-renin ratios and as such, biochemical cure cannot be ascertained. Lastly, extreme heterogeneity was present in this study and although groups were stratified, heterogeneity still persisted.

## Conclusions

The hypertension cure rate of unilateral adrenalectomy in PA was assessed by meta-analysis and the results indicate that the hypertension cure rate is not high. We could not obtain a pooled odds ratio through logistic regression analysis by the method of meta-analysis because of inconsistent data pertaining to risk factors and outcome. Given the limitations of the included studies, particularly detection bias, large-sample clinical trials are required to verify the rationale of potential preoperative predictors in developing a new and effective prediction model.

## Abbreviations

ACI: abdominal calcification index
APA: aldosterone-producing adenoma
ARS: aldosteronoma resolution score
AVS: adrenal vein samplings
BMI: Body mass index
CNKI: China National Knowledge Internet
DBP: diastolic blood pressure
IHA: Idiopathic hyperaldosteronism
LDL: Low Density Lipoprotein
LVM: Left Ventricular Mass
LVMI: Left Ventricular Mass Index
MOOSE: Meta-analysis Of Observational Studies in Epidemiology
PA: primary aldosteronism
PAC: plasma aldosterone concentration
SBP: systolic blood pressure
